# Transparency in Artificial Intelligence Reporting in Ophthalmology-A Scoping Review

**DOI:** 10.1016/j.xops.2024.100471

**Published:** 2024-01-18

**Authors:** Dinah Chen, Alexi Geevarghese, Samuel Lee, Caitlin Plovnick, Cansu Elgin, Raymond Zhou, Eric Oermann, Yindalon Aphinyonaphongs, Lama A. Al-Aswad

**Affiliations:** 1 Department of Ophthalmology, NYU Langone Health, New York, New York; 2 Department of Neurosurgery, NYU Grossman School of Medicine, New York, New York; 3 Health Sciences Library, NYU Langone Health, New York, New York; 4 Department of Ophthalmology, Istanbul University-Cerrahpasa, Istanbul, Turkey; 5 Department of Neurosurgery, Vanderbilt School of Medicine, Nashville, Tennessee; 6 Department of Neurosurgery, NYU Langone Health, New York, New York; 7 Department of Medicine, NYU Langone Health, New York, New York; 8 Department of Population Health, NYU Grossman School of Medicine, New York, New York

**Keywords:** AI, Clinical decision support, AI fairness, AI transparency, Model cards

## Abstract

**Topic:**

This scoping review summarizes artificial intelligence (AI) reporting in ophthalmology literature in respect to model development and validation. We characterize the state of transparency in reporting of studies prospectively validating models for disease classification.

**Clinical Relevance:**

Understanding what elements authors currently describe regarding their AI models may aid in the future standardization of reporting. This review highlights the need for transparency to facilitate the critical appraisal of models prior to clinical implementation, to minimize bias and inappropriate use. Transparent reporting can improve effective and equitable use in clinical settings.

**Methods:**

Eligible articles (as of January 2022) from PubMed, Embase, Web of Science, and CINAHL were independently screened by 2 reviewers. All observational and clinical trial studies evaluating the performance of an AI model for disease classification of ophthalmic conditions were included. Studies were evaluated for reporting of parameters derived from reporting guidelines (CONSORT-AI, MI-CLAIM) and our previously published editorial on model cards. The reporting of these factors, which included basic model and dataset details (source, demographics), and prospective validation outcomes, were summarized.

**Results:**

Thirty-seven prospective validation studies were included in the scoping review. Eleven additional associated training and/or retrospective validation studies were included if this information could not be determined from the primary articles. These 37 studies validated 27 unique AI models; multiple studies evaluated the same algorithms (EyeArt, IDx-DR, and Medios AI). Details of model development were variably reported; 18 of 27 models described training dataset annotation and 10 of 27 studies reported training data distribution. Demographic information of training data was rarely reported; 7 of the 27 unique models reported age and gender and only 2 reported race and/or ethnicity. At the level of prospective clinical validation, age and gender of populations was more consistently reported (29 and 28 of 37 studies, respectively), but only 9 studies reported race and/or ethnicity data. Scope of use was difficult to discern for the majority of models. Fifteen studies did not state or imply primary users.

**Conclusion:**

Our scoping review demonstrates variable reporting of information related to both model development and validation. The intention of our study was not to assess the quality of the factors we examined, but to characterize what information is, and is not, regularly reported. Our results suggest the need for greater transparency in the reporting of information necessary to determine the appropriateness and fairness of these tools prior to clinical use.

**Financial Disclosure(s):**

Proprietary or commercial disclosure may be found in the Footnotes and Disclosures at the end of this article.

As artificial intelligence (AI) becomes more widely available and accepted in clinical settings, it is imperative that clinicians are able to critically appraise models, particularly for algorithmic fairness and appropriate use. Traditionally, reporting guidelines for diagnostic tests and interventions have focused on clinical trial design and outcomes. However, for AI and other predictive models, performance can be highly dependent on model development. Details of algorithm training, alongside clinical trial testing, can inform whether an AI system is generalizable or vulnerable to bias, but reporting of this information is often variable.

In a high stakes domain such as medicine, algorithmic transparency and explainability are necessary to ensure appropriate and equitable use. These principles were emphasized in the Food and Drug Administration (FDA) AI/machine learning (ML) Software as a Medical Device Action Plan, in which the FDA outlined future guidance on good ML practices related to training, interpretability, and evaluation, and other issues such as predetermined change protocols, model transparency, and bias evaluation and elimination.^1^ These standards will be critical for building trust in AI systems before they are employed clinically.

There have been multiple instances of algorithmic bias in health care, exposed after use in clinical settings. In an evaluation of a commercially available risk assessment tool, often used by health systems and payers, Obermeyer et al^2^ found that the algorithm identified Blacks of equal or worse health status for additional services less frequently than their White counterparts. Bias arose from the use of proxy variables that mirrored structural inequalities in health care utilization; prediction of future health needs was based on historical health care utilization (total medical expenditure) costs. In this case, because training data and methodology were proprietary and unavailable, the authors reverse-engineered the algorithm in order to identify the causes of bias.^2^

Similarly, image-based algorithms may be subject to bias, often due to unbalanced data in the broader setting of health care disparities. According to the American Academy of Dermatology, skin cancer is diagnosed at more severe stages in people of color; 16% of melanoma cases in Blacks are diagnosed after metastasis with a corresponding, 5-year relative survival rate of 67% in comparison with 92% among Whites.^3^ In a review of publicly available skin cancer image datasets, 1.3% of images had associated ethnicity data and only 11 of 2436 images were of Fitzpatrick skin type V or higher.^4^ In the setting of unbalanced training data, algorithms for diagnosis of skin cancer run the risk of misclassification for darker skin colors and in doing so, could further perpetuate health care inequity.^5^ Beyond this, AI has been shown to predict race from even low quality images from multiple modalities, independent of proxies (such as disease distribution), where clinicians/radiologists cannot.^6^ These examples highlight the importance of access to information regarding algorithm development when evaluating models.

Ophthalmology has been a leader in AI research; IDx-DR (Digital Diagnostics) was the first ever FDA-approved AI-based device for autonomous diagnosis in any field of medicine.^7^ This was followed shortly by the approval of EyeArt (EyeNuk) for autonomous detection of diabetic retinopathy (DR).^8^ However, just as for other specialties, there are challenges to AI research in ophthalmology. It is often difficult to discern the provenance of the ophthalmic data used to train and validate algorithms either due to their proprietary nature or because publicly available datasets do not report demographic metadata. In a review of all open-access image datasets in ophthalmology, <20% reported any demographic data and over half were collected from clinical trials, which, in the United States (US), suffer from consistent underrepresentation.^9^ While there are many exciting applications for AI in ophthalmology on the horizon, standards for transparency in reporting, especially as it relates to model training and development, should be clarified.

This scoping review characterizes the current state of transparency in reporting AI literature in ophthalmology. In designing this study, we referred to 2 existing AI reporting guidelines, the minimum information about clinical AI modeling (MI-Claim) checklist,^10^ and the CONSORT-AI extension for randomized trials.^11^ We also drew on Mitchell et al’s^12^ paper, “Model Cards for Model Reporting,” to further specify details for the evaluation of algorithmic bias. Briefly, “model cards” contain basic model information including intended use, metrics, details on training and evaluation datasets and ethical considerations, and are meant to resemble nutritional labels in format.^12^ These cards are intended to simplify the process of evaluating and comparing models and promote transparency. We previously suggested the adoption of this tool for ophthalmology, and argued for the inclusion of factors such as reference standards and risks associated with training input data (e.g., the use of imaging reports employing normative databases).^13^ A sample model card for ophthalmology is shown in Fig S2 of the supplement (available at https://www.ophthalmologyscience.org). Using these resources, we compiled a list of reporting factors and evaluated primary research articles utilizing ML algorithms for the prospective classification of ophthalmic conditions.

Our goal in this review is to describe the current landscape of AI reporting in ophthalmology to advocate for more transparent documentation at both the level of model development and prospective clinical testing. We believe promoting transparency at these stages can improve effective and equitable use in clinical settings.

First, we outline some basic themes and terminology related to the concepts referenced throughout this review.

## Basic Terms and Concepts

### Training Data

Training data is used to train the model. In supervised learning (all models evaluated in this review are supervised), training data is labeled, often annotated by ophthalmologists. This labeled data constitute the “ground truth.”

### Testing Data

Testing data is used to evaluate the model performance. The model does not “learn” from this dataset. While the source of this data is the same as training data, test sets should be kept separate from training sets during the development process.

### Ground Truth

Ground truth represents the verifiable state of the input data. For example, in the case of a model for the classification of DR from fundus photos, the ground truth is established by training labels that indicate the presence or absence of DR and/or signs of DR.

### External Validation

We refer to external validation of models as the testing of models on datasets that are sampled from disparate populations relative to the original training/testing data. This may be conducted using retrospective data (e.g., use of a publicly available dataset such as Messidor-2 or EYEPACS) or prospectively, through observational or clinical trial testing.

### Reference Standards

Performance metrics of the model are calculated by comparing model performance against a reference standard, which usually represents the gold standard for diagnosis where one exists. This may differ by country (e.g., international clinical DR vs. EURODIAB grading criteria).

### Data Governance

A broad term for issues related to the management and regulation of data used for AI model development.

### Data Provenance

This encompasses questions related to the origins of data such as who collected it, and for what purpose. This includes metadata of the datasets and model. Data provenance is important for assessing AI fairness and replicating or comparing models using differing datasets.

### AI Fairness

Though there is no agreed-upon definition for fairness, this generally focuses on inclusivity through an assessment of training datasets for potential bias, testing for bias, evaluation of disparities in model performance, and consideration of the characteristics of teams developing models and populations that will or will not have access to the model.

### Explainability

Includes elements of transparency including training/testing data information, performance metrics with consideration of risks/benefits (e.g., screening thresholds), measures to overcome the “black box” phenomena such as saliency mapping of regions of interest, and communication of limitations and protocols related to accountability.

The parameters we assess in this review relate to the above concepts, many of which are not mutually exclusive. Specific factors are further defined in the methods section.

## Methods

In this scoping review, we sought to answer whether AI studies in ophthalmology report both basic details regarding models such as model architecture and performance metrics, and particulars of their data at the training and external validation stages of testing including aspects of data provenance and demographics such as age, race and/or ethnicity, and gender. All of these factors play a role in understanding the appropriate scope of use of these models. As our search method included a broad range of studies across multiple subspecialties in ophthalmology, a traditional systematic review and meta-analysis was not pursued. We focus only on prospective validation studies in order to highlight how information on models that have undergone the most advanced validation testing (both retrospectively and prospectively) is reported.

### Eligibility Criteria

Prospective clinical trials and observational studies evaluating AI models for disease classification or progression of ophthalmic conditions were considered for full-text review. All ophthalmic conditions and input types (e.g., fundus images, OCT images, clinical data, external photos, etc.) were included.

We excluded studies that:

- Are retrospective in study design, not associated with a prospectively validated model
- Studies without outcomes of interest such as models with intended use other than for classification of disease or progression of disease (e.g., models with intended use of segmentation of images)
- Are models trained using nonhuman related data
- Are not peer-reviewed (e.g., abstracts, editorial commentaries, and letters)
- Do not report primary results (e.g., review articles)
- Do not have an English translation

### Search Strategy

This review was performed in accordance with the Preferred Reporting Items for Systematic Reviews and Meta-Analysis (PRISMA) extension for scoping reviews. We conducted a comprehensive literature search using PubMed, Medline (Ovid), EMBASE (Ovid), Web of Science, and CINAHL in January 2022, using keywords and subject headings for AI, ophthalmology, and ophthalmic conditions. Results were limited to primary research articles published between October 2011 and January 2022 as the majority of research was published during this period. Manual searches for related references from articles included in the full-text review were also performed. We found that in many cases, groups may have published training and retrospective validation studies prior to conducting and publishing on the use of that same model, prospectively. We chose to include these associated, previously published articles on training and retrospective validation of these prospectively validated models where available, in an effort to provide the most comprehensive evaluation at the level of the AI model itself. If data could not be found through this method, we reviewed websites for models where applicable. Full search details can be seen in Table S1 of the supplement (available at https://www.ophthalmologyscience.org). Databases were last searched in January 2022. Studies that were not associated with prospective validation of models were excluded.

All references were imported into Covidence (Melbourne, Australia), a management tool for systematic reviews. Titles and abstracts were independently assessed by ≥2 reviewers using Covidence (A.G., S.L., C.E., and R.Z.). The reviewers included or excluded articles on the basis of the inclusion and exclusion criteria. A third reviewer adjudicated any disagreements between the 2 reviewers (D.K.C.). Unanimously approved studies and those included following resolution of disputes were advanced to full-text review. Studies were assessed for eligibility for extraction according to inclusion and exclusion criterion, before proceeding to data extraction. Funding sources for all included studies are listed in Table S2 of the supplement (available at https://www.ophthalmologyscience.org).

### Data Extraction

Data from included studies were extracted independently by 2 reviewers (D.K.C. and A.G.) using Covidence and all studies were then reviewed for a second time by a single reviewer (D.K.C.). Any discrepancies were resolved through consensus. Summary statistics were calculated in Excel (Microsoft).

We extracted elements related to transparency (derived from model cards, MI-CLAIM checklist, and CONSORT-AI guidelines). These elements are listed in Table 3 with brief definitions, examples and assumptions where applicable.

**Table 3:** Elements Extracted From Manuscripts in Covidence

Model Details	
Organization developing model	
Model date/version	
Intended use	
Primary intended uses	What does this model achieve? i.e., classification of DR
Primary intended users	Who should this model be applied to? And who should be using this model? i.e., primary care providers
Scope of use∗	In what context should the model be used? i.e., screening of referable DR in diabetics by nonophthalmologists. This should include both intended use and users but further specifies applicable conditions (e.g., setting). Is this explicitly stated or implied?
Inputs	Data that will be analyzed by the model and manufacturer source if imaging device is used, i.e., fundus photos, OCT macula, etc. Are models trained with data derived from device outputs that rely on normative databases?
Outputs	ex. referable DR, closed angle, cataract severity, etc.
Model Architecture	ex. Inception V3, Resnet, etc.
Training data	
Source	Where does the training dataset come from? i.e., publicly available dataset, institution outpatient clinic, nation-wide screening programs
Demographics	Age
	Gender
	Race/Ethnicity
Distribution	ex. number of training images with no DR, mild DR, severe DR, DME, etc., allows for the assessment of dataset balance
Annotation	How were the training images labeled and who did the labeling?
External evaluation data	Both retrospective and prospective/observational
Source	Dataset origin
Demographics	Age
	Gender
	Race/Ethnicity
References standards	What standard was used to judge against model performance? i.e., ophthalmologist evaluation, fundus photo reading center, etc.
Performance measures	
Metrics	Sensitivity, specificity, PPV, NPV, imageability (if applicable)
Decision Thresholds	How are sensitivity/specificity thresholds set according to intended use?
Limitations∗	
Ethical considerations	
Risk/bias mitigating measures	Did the developers report the use of specific measures to improve the generalizability or explainability of their models at the training stage (i.e., augmentation, early stopping, heatmapping/CAM/saliency mapping)
Input imaging risks∗	Did the authors explicitly state or imply any possible risks associated with training data? i.e., unbalanced data
Intersectional testing	Was the model tested with datasets that differed from training data in terms of demographics? May depend on intended use.
Potential inappropriate use cases∗	Did the authors explicitly state or imply certain out-of-scope use cases? i.e., models for referable DR that should not be used for DME identification, models specific to certain populations

DME = diabetic macular edema; DR = diabetic retinopathy; NPV = negative predictive value; PPV = positive predictive value.

∗ There may be significant overlap in reporting of scope of use, input risks, inappropriate use cases and limitations and in many cases this may be implied. If these elements were easily inferred, they were marked as fulfilled by reviewers.

A few articles assessed outcomes of the same model (e.g., IDx-DR, EyeArt, Medios AI) in different settings or populations.^14^, ^15^, ^16^, ^17^, ^18^, ^19^, ^20^, ^21^, ^22^, ^23^, ^24^, ^25^, ^26^ In these cases, we referred to the same training and retrospective validation studies where possible/available in order to characterize algorithms at the model level, not only at the level of the clinical validation study.^27^, ^28^, ^29^, ^30^, ^31^, ^32^, ^33^, ^34^, ^35^, ^36^, ^37^ Parameters specific to clinical validation were evaluated for each individual study, as these studies may not have the same aims or intended uses as described in training and retrospective validation of unique models (i.e., validation using smartphone fundus images or portable cameras, validation in the pediatric population, etc.).

### Data Analysis

The reporting of parameters listed above were summarized and described qualitatively.

## Results

**Figure 1:**
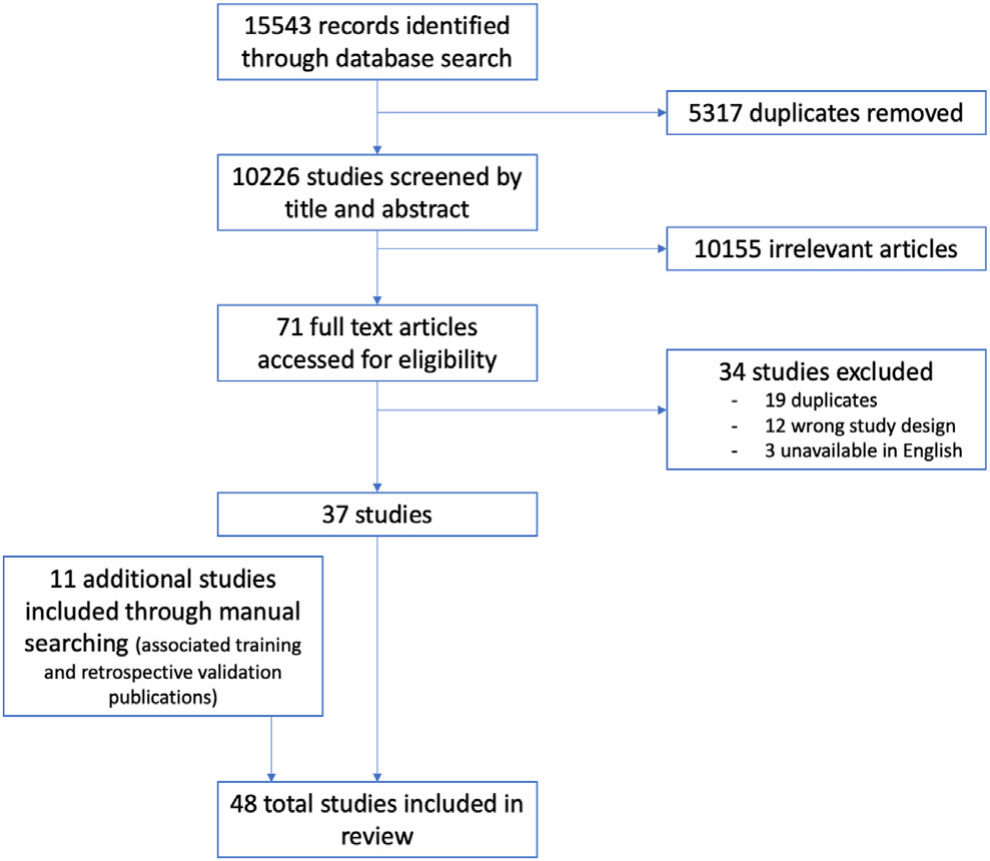
Preferred Reporting Items for Systematic Review and Meta-Analyses Flow Diagram. Fifteen thousand five hundred and forty-three records were identified through a search of PubMed, Medline (Ovid), EMBASE (Ovid), Web of Science, and CINAHL. Of these, 10 226 studies were screened by title and abstract for inclusion and exclusion criteria. Thirty-seven of the 71 full text articles assessed for eligibility were included. Eleven additional studies were included through a manual search for associated training and retrospective publications.

A total of 15 543 studies were imported for screening and 5317 duplicates were removed. The remaining 10 226 studies were included in title and abstract screening and 71 studies were moved to full-text review for evaluation with strict inclusion and exclusion criteria. Thirty-four studies were excluded due to duplication, wrong study outcome (i.e., studies with a primary outcome of imaging segmentation, not disease classification or progression) or design (i.e., retrospective), or unavailable in English. The majority of studies that were excluded conducted evaluation of models using retrospective data only. Thirty-seven prospective observational and clinical trial studies were included and 11 previously published studies related to training or retrospective external validation of those prospectively validated models were added from manual searches based on references. While a total of 48 studies underwent full text extraction, the 11 additional studies were grouped with their corresponding prospective observational studies or clinical trials and analyzed together. Figure 1 shows the Preferred Reporting Items for Systematic Review and Meta-Analyses (PRISMA) flow diagram for our selection criteria. A list of all included studies is provided in Table 4 as well as associated prior publications on model development and retrospective external validation.

**Table 4:** Studies Included in Review Along With Associated Training and Retrospective Validation Studies

Named Models	Country Developed	Authors	Associated Studies∗		Input Type	Primary Output
			Training	Retrospective External Validation		
Airdoc	China	He 2020^38^			Standard Fundus Photo	Any DR
Bosch DR	USA	Bawankar 2017^39^			Handheld Fundus Camera Photo	Any DR
CC-Cruiser	China	Lin 2019^40^		Long 2017^31^	Slit Lamp Photo	Cataract
CARE	China	Lin 2021^41^			Standard Fundus Photo	Multiple retinal diseases
DeepDR	China	Dai 2021^42^			Standard Fundus Photo	Any DR
EyeArt	USA	Olvera-Barrios 2021^20^	Bhaskaranand 2016^29^	Bhaskaranand 2019^34^	Standard Fundus Photo	Any DR
		Liu 2020^18^			Standard Fundus Photo	RDR
		Ipp 2021^16^			Standard Fundus Photo	RDR
		Rajalakshmi 2018^21^			Smartphone Fundus Photo	RDR
		Heydon 2020^15^			Standard Fundus Photo	RDR
		Sarao 2020^22^			Standard Fundus Photo	RDR
IDx-DR	USA	Van der Heijden 2018^25^	Niemeijer 2009^27^	Abramoff 2016^28^	Standard Fundus Photo	RDR
		Wolf 2021^26^			Standard Fundus Photo	RDR
		Abramoff 2018^14^			Standard Fundus Photo	RDR
EyeGrader	China	Keel 2018^43^			Standard Fundus Photo	RDR
EyeWisdom	China	Ming 2021^44^			Standard Fundus Photo	RDR
iGrading	UK	Soto-Pedre 2015^45^			Standard Fundus Photo	Any DR
iHealthScreen	USA	Bhuiyan 2021^46^			Standard Fundus Photo	RDR
Medios AI	Singapore	Sosale 2020^23^			Smartphone Fundus Photo	RDR
		Sosale 2020^24^			Smartphone Fundus Photo	RDR
		Jain 2020^17^			Smartphone Fundus Photo	RDR
		Natarajan 2019^19^			Smartphone Fundus Photo	RDR
Pegasus	UK	Rogers 2020^47^			Smartphone Fundus Photo	RDR
Verisee	China	Li 2021^48^	Hsieh 2021^37^		Standard Fundus Photo	RDR
VoxelCloud Retina	USA	Zhang 2020^49^			Standard Fundus Photo	RDR

Unnamed Models	Country	Authors	Training	Retrospective External Validation	Input type	Primary Output
	China	Wu 2019^50^			Slit Lamp Photo	Cataract
	China	Scheetz 2021^51^	Li 2018^32^		Standard Fundus Photo	RDR
	Singapore	Bellemo 2019^52^			Standard Fundus Photo	RDR
	Singapore	Porporato 2021^53^	Fu 2019^35^		Anterior Segment OCT	Angle Closure
	Brazil	Shigueoka 2018^54^			Clinical data (OCT, SAP parameters)	Glaucoma
	China	Yang 2020^55^			External Photos	Myopia
	Australia	Kanagasingam 2018^56^			Standard Fundus Photo	Any DR
	India	John 2019^57^	John 2016^30^		Standard Fundus Photo	Any DR
	USA	Gulshan 2019^36^ (Google)	Gulshan 2016^61^		Standard Fundus Photo	RDR
	China	Hong 2021^58^			Slit Lamp Photo, Standard Fundus Photo	Multiple causes of vision impairment
	Japan	Nakahara 2021^59^	Shibata 2018^33^		Smartphone Fundus Photo	Glaucoma (smartphone camera)
	India	Pawar 2021^60^			Portable Fundus Camera Photo	RDR

AI = artificial intelligence; DR = diabetic retinopathy; OCT = Ocular coherence tomography; RDR = referrable diabetic retinopathy; SAP = static automated perimetry; UK = United Kingdom; USA = United States of America.

∗ If training and retrospective validation data was not provided in the same clinical trial manuscript, we referred to citations and prior publications on the model.

### Unique Model Characteristics

There were 27 unique models assessed across the 37 studies. Seventeen of 27 models were developed by Asian institutions or companies, 6 by North American institutions or companies, 2 by European institutions, 1 by an institution in Australia, and 1 was developed by an institution in Brazil.

Two of 27 studies did not report model version and this could not be determined using other sources. Two models were ML; 1 study reported the use of multiple ML classifiers, while the other did not specify the ML model used. Twenty-three studies were deep learning models: 20 were convolutional neural network models; the remaining 2 were un-specified deep learning models. Two did not report whether the model was deep learning or ML. The specific model architecture used (i.e., Resnet, Inception V3) for 6 models was not identifiable.

### Clinical Validation Study Characteristics

#### Countries Where Studies Were Conducted

Of the 37 observational studies and clinical trials that were included, 5 continents were represented. Twenty-one studies were conducted in Asia, predominantly in China and India with 10 and 9 studies, respectively. Six studies were conducted in North America, 5 in the US and 1 in Mexico. Five studies were conducted in Europe, 3 in Australia,^56^, ^43^, ^51^ and 1 was conducted in South America and Africa, respectively.

#### Inputs of Clinical Studies

The majority of study inputs were fundus photos (32/37). Of these studies, 23 were standard 45 degree fundus photos, 7 were smartphone fundus photos, and 2 studies were conducted using a portable/handheld fundus camera (Bosch DR, IntucamPrime).^39^^,^^60^ In some cases where training information was available, studies evaluated the use of smartphone photos with a model that was trained using standard fundus photos. Other studies utilized models with inputs of anterior segment OCT images, slit lamp photos, external photos, and clinical data (OCT, Humphrey visual field, FDT parameters).

Only 3 studies did not report the specific manufacturer of the imaging devices used for inputs; they were all studies utilizing slit lamp photos. A list of manufacturers of imaging devices used in clinical studies is provided in Table S5 of the supplement (available at https://www.ophthalmologyscience.org). Ten studies evaluated the use of images from >1 imaging device manufacturer.

#### Outputs

The vast majority of unique AI models were for DR (18/27). There was a wide variety in outputs for DR and various definitions for “referable” disease; in some studies, diabetic macular edema (DME) was included in the referable case definitions. Two models addressed classification problems related to glaucoma. The remaining 7 models focused on myopia, cataract, multiple retinal diseases, visual impairment, and angle closure.^53^ Table 4 details diseases, as well as input data types for all studies.

#### Primary Intended Uses

All studies reported primary intended use, and all models were classification models for the diseases listed in Table 4. Fifteen of the total 37 clinical studies did not explicitly define or imply the primary intended users.

#### Scope of Use

The scope of use was determinable in 7 of 37 studies. Based on our definition for this criterion, studies had to report both intended use and users and additionally report the setting/environment for which the model is suited.

### Training Data Study Characteristics

Training level characteristics are shown in Table 6. We were able to find training information for 27 of the total 37 studies,^14^^,^^17^^,^^19^^,^^23^, ^24^, ^25^, ^26^^,^^36^^,^^56^, ^43^, ^51^, ^39^^,^^53^, ^45^, ^54^, ^52^, ^57^, ^40^, ^50^, ^55^, ^49^, ^42^, ^58^, ^48^, ^41^, ^44^, ^59^ 22 of which were unique AI models. Training information could not be found for the remaining 5 unique models.

**Table 6:** Training, Retrospective External Validation, and Prospective Validation Characteristics

Model Training			Retrospective External Validation			Observational Studies or Clinical Trials		
Source of training data			Source of retrospective external validation data			Source of prospective validation data		
China		8	France, UK		2	Asia		
China, India		1	USA		2		China	10
China, USA		1	USA, China		1		India	9
China, Australia		1	USA, France, UK		2		Singapore	1
India		1	Australia, Singapore		1		Japan	1
Singapore		1	Japan		1	North America		
Japan		1	India		3		USA	5
USA		1	Unspecified		1		Mexico	1
USA, India		3				Europe		
USA, Australia		1					UK	2
Netherlands		1					Netherlands	1
Brazil		1					Spain	1
							Italy	1
						Africa		
							Zambia	1
						Australia		
							Australia	3
						South America		
							Brazil	1
Training Dataset Size (# of images)		**22/27**	Training Dataset Size (# of images)		13/13	Study size∗ (# of patients)		**37/37**
	Average Size	82 899.24		Average Size	102 157.17		Average Size	3227.61
	Median Size	39 136		Median Size	7693		Median Size	526
	Range	886–666 383		Range	53–850 908		Range	50–47 269
Average age		**7/27**	Average Age		5/13	Average Age		**29/37**
Gender		**7/27**	Gender		5/13	Gender		**28/37**
	Avg Male%	46.23		Avg Male%	47.64		Avg Male%	51.66
	Avg Female %	52.63		Avg Female %	52.36		Avg Female %	48.34
Race/Ethnicity		**5/27**	Race/Ethnicity		2/13	Race		**9/37**
Training study (corresponding prospective validation study)	Li 2021^48^	Chinese 100%	External validation study (corresponding prospective validation study)	Long 2017^31^ (Lin 2019)^40^	EyePACS data	Liu 2020^18^ (EyeArt)		White 15%
	Zhang 2020^49^	APTOS dataset			White 5%–10%			Black 78.3%
		White 7.6%			Black 5%–10%			Other 6.7%
		Black 4.3%			Asian 5%–10%	Ipp 2021^16^ (EyeArt)		White 74%
		Asian 5%			Hispanic 55%			Black 18%
		Hispanic 50.2%			Other 5%–10%			Asian 2%
		Other 32.8%		Li 2018^32^ (Scheetz 2021)^51^	(Reported only ethnicity)			Hispanic 22%
	Fu 2019^35^ (Porporato 2021^53^)	(Reported only ethnicity) Chinese, Indian, Malay 100%			Indigenous Australians			Other 6%
	Shigueoka 2018^54^	White 75.7%			Malays	Heydon 2020^15^ (EyeArt)		White 51%
		Black 24.3%			White Australians			Black 14.2%
	Gulshan 2016^61^	Partial (EyePACS data only)						Asian 19.6%
		White 5%–10%						Other 13.8%
		Black 5%–10%				Sarao 2020^22^ (EyeArt)		White 100%
		Asian 5%–10%				Wolf 2021^26^ (IDx-DR)		White 57.1%
		Hispanic 55%						Black 32.3%
		Other 5%–10%						Hispanic 4.2%
Distribution over various factors		10/27						Other 6.5%
Descriptions of Ground Truth		18/27				Abramoff 2018^14^ (IDx-DR)		White 63.4%
								Black 28.6%
								Asian 1.6%
								Hispanic 16.4%
								Other 6.4%
						Zhang 2020^49^ (VoxelCloud Retina)		(Reported only ethnicity)
								Chinese 96.1%
						Porporato 2021^53^		(Reported only ethnicity)
								Chinese 82.1%
								Non-Chinese 17.9%
						Shigueoka 2018^54^		White 47.5%
								Black 28.2%
								Asian 0.8%
								Other 23.3%

UK = United Kingdom; USA = United States of America.

∗ One study reported number of images, not patients and is not included in these summary statistics (Lin et al, 2021).^27^

Models reporting on training and development sourced their data from China for 8 models, Singapore for 2 models, India for 1 model, Japan for 1 model, and multiple countries for 7 models. Two models used training data from Europe and South America. One study did not report the source of training data. Seven studies used publicly available data either alone or in combination with proprietary data. All 22 studies reported the size of their training datasets, which averaged 82 900 images. The median number of images in training datasets was 39 136, with an interquartile range (IQR) of 68 100, min of 866, and max 600 000.

#### Training Data Demographics

Seven studies reported the age of patients. Among the 5 studies reporting averages, the average age of participants was 60 ± 1.88%. One study reported an age range only (6–18),^55^ another reported that > 92.7% of patients were over the age of 18.^50^ Seven studies reported the gender of patients, averaging 46.2 ± 2.02% male and 52.6 ± 1.72% female (remaining unknown). Five studies reported race and/or ethnicity. Among these studies, 3 enrolled patients of White race/ethnicity with a median of 31.1% (min 7.6%, max 75.7%, IQR 34.05).^36^^,^^54^^,^^49^ Three studies included patients of Black race/ethnicity with a median of 12.86% (min 4.3%, max 24.3%, IQR 10).^54^^,^^49^ Three studies enrolled patients of Asian race/ethnicity with a median of 38.33% (min 5%, max 100%, IQR 47.5).^36^^,^^53^^,^^49^ Two studies enrolled patients of Hispanic ethnicity at 50.2% and 55% of total study population.^36^^,^^49^ One study reported only the ethnic makeup of their population.^48^

Among these studies, average percentage of White race/ethnicity patients was 31.1% (± 22.3%), Black 12.87% (± 5.9%), Asian 38.3% (± 30.87%), and Hispanic 52.6% (± 2.4%). One study reported only the ethnic makeup of their population.^48^

#### Training Data Distribution

Ten of the 27 studies reported the distribution over various factors of their datasets. For example, Bellemo et al^52^ reported a training dataset composition of 36 109 images with no or mild nonproliferative DR and 2055 images with referable DR (597 moderate nonproliferative DR, 478 severe nonproliferative DR, 70 proliferative DR, and 2026 images with DME).

#### Training Data Annotation Description (Ground Truth)

Eighteen studies reported on the annotation process for training images, including a description of who performed annotations (i.e., an ophthalmologist, senior graders, etc.).

### Retrospective External Validation

Table 6 details retrospective external validation characteristics. Thirteen of 27 unique models had ≥1 retrospective external validation study either published conjointly with the clinical study or in previous publications. For models with multiple separate external validation studies, we chose only 1 study for evaluation. Nine of these studies used datasets that are now publicly available for validation, either in conjunction with other datasets or standalone. These datasets include: Messidor (France, United Kingdom), EyePACS (US), IDRiD (India), and the APTOS Blindness Detection dataset (India). Private datasets came from India, China, Singapore, Australia and Japan. One dataset was developed using web searches on Google, Baidu, and Bing and the provenance of this dataset is unclear.

#### Demographic Characteristics

All 13 studies reported the number of images in their datasets, with a median of 4092 (min 53, max of 850 908 images, IQR of 28 397.5). Five studies reported the ages of patients in their datasets and gender distribution, each. Two studies reported the race and/or ethnic makeup of their datasets. One study reported 5% to 10% of patients in the study were White, 5% to 10% Black, 5% to 10% Asian, and 55% Hispanic. One study reported only ethnicities.

#### Reference Standards

Nine of 13 studies reported reference standards for their retrospective external validation studies.

### Clinical External Validation (Observational Studies and Clinical Trials)

Table 6 shows characteristics of the observational studies and clinical trials. Nine studies evaluated FDA-approved devices, EyeArt and IDx-DR, in different settings. One study evaluated EyeArt for use with smartphone fundus photos in India, another for use with images from an LED confocal scanner in Italy. Other locations included the United Kingdom and US. IDx-DR was evaluated in the US and in the Netherlands. It was also evaluated for the pediatric population. One algorithm was evaluated across 4 different included studies, Medios AI, which is an offline DR grading AI that utilizes smartphone fundus images obtained on the Remidio FOP. All 4 studies were conducted in India at different levels of care (dispensaries, outpatient departments, tertiary care centers).

The remaining studies focused on a variety of conditions (Table 4), and the majority were conducted in Asia. Only 1 study was conducted in Zambia, Mexico, and Brazil, respectively.

#### Study Population Characteristics

All but 1 of the 37 studies reported the number of enrolled patients. This study reported the number of photos evaluated but did not report the total number of patients. The median study size was 526 patients, IQR of 1179.25, and ranged from 50 to 47 269 patients.

#### Demographics

Twenty-nine of 37 studies reported the ages of enrolled and 28 reported gender. On average, 51.6 ± 1.35% of patients were male and 48.34 ± 1.35% were female. Nine studies reported the racial or ethnic composition of their study populations. Table 6 lists these specific studies.

Among these 9 studies, 2 reported only ethnicity data. Of the 7 studies reporting race, the mean demographic breakdown among studies reporting race was White 58.3 ± 9.83%, Black 28.5 ± 9.28%, and Asian 3.4 ± 2.71%. The mean for Hispanic ethnicity was 6.0 ± 3.49%. Mean for other race/ethnicity was 31.1 ± 13.09%.

#### Reference Standards

Thirty-six of the 37 studies clearly stated the reference standards against which the model was evaluated. Only 1 study did not provide clear information on reference standards. In this study, prospective testing was done in a subset of patients (n = 1294) to assess improvement in model accuracy following real-time image quality feedback by the algorithm. While they reported performance metrics following feedback implementation (repeating photos for images that were of poor quality), the reference standard for this analysis was not stated.

#### Decision Thresholds

Thirteen of 37 studies reported decision thresholds for their models.

#### Performance Metrics

For studies reporting >1 output, we evaluated the performance metrics for the primary outcome, where possible. For example, many DR studies reported outcomes for “referable DR” and “any DR.” In these cases, “referable DR” was designated the primary output. For the 2 studies using models for multi-disease classification, the first reported condition was evaluated.

All 37 studies reported ≥1 of the following metrics: area under the curve, sensitivity, specificity, positive predictive value (PPV), negative predictive value (NPV), and imageability. Thirty-two studies reported sensitivity, specificity, PPV, and NPV, or could be calculated from the information provided. Twenty studies reported area under the curve, and 22 studies reported imageability. Disease prevalence was reported by all but 2 studies. A summary of averages of these metrics are provided in Table 7.

**Table 7:** Summary Performance Metrics of Observational Studies and Clinical Trials

Performance Metrics	# Performance Metrics Reported	Average∗	Range∗
AUC	20	0.90	0.67–0.986
Sensitivity	36	90.16%	37%–100%
Specificity	35	84.63%	46%–98.79%
PPV	18	51.53%	9%–94.4%
NPV	16	92.21%	95%–100%
Imageability	22	87.98%	44.6%–100%
Prevalence	35	24.24%	1%–68.7%

AUC = area under the curve; NPV = negative predictive value; PPV = positive predictive value.

∗ Studies may have set different thresholds depending on context of use (e.g., screening). In many cases, studies reported > 1 outcome. Averages and ranges are for primary output performance.

#### Intersectional Testing

Thirteen of 37 reported intersectional testing, or validation of their studies in diverse populations that differed from training populations.

#### Limitations

Six studies did not report any limitations in their study designs or applications.

#### Ethical Considerations

We evaluated ethical considerations at the level of training as well as elements reported in the clinical testing studies. Sixteen of the 27 unique models reported using risk-mitigating measures in the development of their models to improve generalizability such as augmentation, and/or explainability of their models with saliency mapping. Ten studies specifically mentioned certain risks associated with inputs of their training set, such as limited racial/ethnic diversity.

At the level of the prospective clinical validation, 28 studies reported potential inappropriate use cases for their models. For example, multiple studies using classification models for DR remarked that DME could not be reliably determined or that their models were not suitable for identifying peripheral retinal disease. Others commented on the need for additional testing in specific populations (community-based) or stressed that the model should be used as a clinical decision support tool, in conjunction with evaluation by an ophthalmologist, given a high false-positive rate.

### Summary of Transparency Parameters

For the 23 prospective studies reporting retrospective external validation testing, we evaluated 36 transparency parameters. Among these studies, the average number of parameters reported was 23.7 (± 0.79), with a range of 14 to 29. Table 8 details number of parameters reported by the study.

**Table 8:** Summary of Transparency Parameters by Study

Named Models	Authors	# Transparency Parameters Reported (Listed in Table S1, Based on MI-CLAIM, CONSORT-AI, and Ophthalmology Model Card)	% Reported
Airdoc∗	He 2020^38^	18	58.06%
Bosch DR∗	Bawankar 2017^39^	20	64.52%
CC-Cruiser	Lin 2019^40^	23	63.89%
CARE	Lin 2021^41^	21	58.33%
DeepDR	Dai 2021^42^	25	69.44%
EyeArt	Olvera-Barrios 2021^20^	17	47.22%
	Liu 2020^18^	27	75.00%
	Ipp 2021^16^	25	69.44%
	Rajalakshmi 2018^21^	21	58.33%
	Heydon 2020^15^	25	69.44%
	Sarao 2020^22^	23	63.89%
IDx-DR	Van der Heijden 2018^25^	27	75.00%
	Wolf 2021^26^	29	80.56%
	Abramoff 2018^14^	26	72.22%
EyeGrader∗	Keel 2018^43^	21	67.74%
EyeWisdom∗	Ming 2021^44^	19	61.29%
iGrading∗	Soto-Pedre 2015^45^	18	58.06%
iHealthScreen	Bhuiyan 2021^46^	14	38.89%
Medios AI	Sosale 2020^24^	25	69.44%
	Sosale 2020^23^	22	61.11%
	Jain 2020^17^	23	63.89%
	Natarajan 2019^19^	22	61.11%
Pegasus	Rogers 2020^47^	23	63.89%
Verisee∗	Li 2021^48^	21	67.74%
VoxelCloud Retina	Zhang 2020^40^	27	75.00%

Unnamed Models	Authors	# Transparency Parameters Reported	% Reported
	Wu 2019^50^	18	58.06%
	Scheetz 2021^51^^,^∗	29	80.56%
	Bellemo 2019^52^	26	83.87%
	Porporato 2021^53^	27	87.10%
	Shigueoka 2018^54^	26	83.87%
	Yang 2020^55^	20	64.52%
	Kanagasingam 2018^56^	19	61.29%
	John 2019^57^^,^∗	18	50.00%
	Gulshan 2019^36^ (Google)∗	29	80.56%
	Hong 2021^58^	15	48.39%
	Nakahara 2021^59^^,^∗	24	66.67%
	Pawar 2021^60^	15	48.39%

∗ No retrospective external validation studies available, transparency parameters out of 31. All other studies out of 36 parameters.

For the remaining 14 studies without retrospective external validation, we evaluated 31 total parameters. The average number of parameters reported was 20.2 (±3.78), with a range from 15 to 27. The studies with the lowest and highest percentage of parameters reported were Bhuiyan et al and Porporato et al, respectively. Table 8 shows the number and percent of transparency parameters reported by the study.

#### Most Reported Parameters

All studies reported intended use, input, and output. Nearly all observational and clinical trial studies reported ≥1 of the following performance metric: sensitivity, specificity, PPV, and NPV. Only 1 study did not report sensitivity.

#### Least Reported Parameters

The least likely parameters to be reported for all study types (training, retrospective validation, and clinical validation) were the racial and/or ethnic demographics of their datasets. At the training level, only 5 studies reported race and among external validation studies, and 2 reported race. Only 9 observational and clinical trial studies reported race and/or ethnicity; 6 of these studies evaluated the use of FDA-approved commercially available models, EyeArt and IDx-DR.

Scope of use was similarly difficult to ascertain from a majority of the studies; only 7 of 37 met our reporting definition for this parameter.

## Discussion

Over the last decade, AI research in ophthalmology has grown rapidly; we found >10 000 related articles, the majority of which were published within the last 5 years. In 2020, the SPIRIT-AI and CONSORT-AI guidelines were published, 2 years after the seminal FDA-approval of IDx-DR for autonomous diagnosis of DR. While these clinical trial guidelines are essential, there is a significant gap in guidance regarding reporting at the level of model development.

Transparency in AI reporting is critical when evaluating models for possible unintended bias and determining generalizability to specific populations for implementation in clinical settings. Models may not be generalizable across demographics (a model designed for DR detection in adults may not be as accurate in the pediatric population) or settings (countries may employ different diagnostic criteria for the same disease).^62^ Applying models in the wrong context may lead to misclassification, and when done consistently, to inequitable health outcomes at scale. For providers, determining the appropriate context of use requires an understanding of the development and validation of these models. However, this information can be difficult to ascertain. In this setting, we have previously advocated for the adoption of a tool to promote transparency and AI fairness,^13^ model cards, which we believe can play a role in streamlining not only reporting, but also the process of appraising and comparing models in ophthalmology.

On a broader level, this information allows us to visualize the growing divide among countries with access to AI development and use and brings up questions related to AI colonialism. Currently AI research is limited to institutions with access to the vast computational resources required for such work, creating a “compute divide.”^63^ In this review, we found that most models were developed in Asia and some from Europe and the US. Only 1 was trained using data from South America and none were developed in Africa. Similarly, most clinical trial testing was performed in the US, Europe, and Asia. As AI becomes more prevalent and, in many cases, outperforms traditional methods of screening and diagnosis, we may begin to see not only a growing computational divide, but also worsening of health inequity. We must consider who produces models, for whom they are made, and which patient populations have access to them. Reporting guidelines lay the foundation for this understanding.

In this scoping review, we describe the landscape of reporting in AI literature in ophthalmology. We included only models that have undergone prospective validation testing and the prior studies published on these same models related to retrospective training and validation to demonstrate the level of transparency of reporting from model development through clinical trial. Our aim is to understand what elements related to fairness are reported, at both the level of prospective validation and model development, and to demonstrate the need for greater transparency at all levels. Using the model card we adapted for ophthalmology as a guide, along with the CONSORT-AI and MI-CLAIM recommendations, we found that there was a wide range in the reporting of both basic details of algorithm training and evaluation. This has implications for bias, equity, and the overall scope of use of models.

### Training Data

Among the >10 000 articles we screened, we found 37 observational studies and clinical trials that evaluated the use of 27 unique AI models for the classification of ophthalmic conditions. We were able to find information on training data for 22 of the 27 unique models. For the remaining 5 models, no training information could be located. All 22 of the studies reported both the source and size of their training datasets but there was limited reporting of the other factors we evaluated at the training level, notably demographics, ground truth annotations, and data distribution.

#### Demographics

Though we were able to identify the source of training data for many of the models, few studies reported the demographics of this data. Only 7 studies reported age and gender, and only 5 studies reported race and/or ethnicity. Algorithmic bias may be unintentionally introduced if the demographic composition of the training dataset does not align with the context of its intended use. For example, fundus pigmentation is known to vary with race; algorithms trained on a dataset of light funduses may underperform when tested on patients with darker funduses.^64^

Though there are clear guidelines for the reporting of demographic information at the level of clinical trials (SPIRIT- AI, CONSORT-AI), this information is often left out in the reporting of training level data. The MI-CLAIM checklist guidelines, specifically for model development, recommend the reporting of population composition in order to determine how representative groups are of clinical settings. In countries like the US with heterogeneous populations, the use of AI should be validated for a representative patient population. Access to training data demographic information may help providers judge whether models should be used for their patients.

#### Ground Truth

The annotation process for training data is also important for understanding model fairness, as it establishes the ground truth. Eighteen of 27 models described the annotators of the training images (i.e., residents, ophthalmologists, and trained graders). Understanding who provided this information and how or if it was adjudicated gives insight into the validity of the model.

Though no studies in this review utilized OCT reports as inputs, OCT-based AI is on the horizon (Notal, EyeNuk). It is important to note that OCT reports rely on normative databases that may not be demographically representative. For example, Heidelberg Spectralis’ retinal nerve fiber layer thickness report is founded on the values of White normal subjects.^65^ A reference database with racial and ethnic diversity is available for purchase as a separate license. Because macular and retinal nerve fiber layer thickness is known to vary by race, the use of these reports as inputs for model development may inadvertently introduce bias.

#### Data Distribution

Even fewer studies (10/27) reported the distribution over factors of their training data (i.e., number of images with mild, moderate, or severe disease). This element is critical for understanding dataset balance, and the model’s ability to accurately classify the full spectrum of disease.

### External Validation, Retrospective and Prospective Studies

#### Demographics

Similarly to training data, we found that the reporting of demographic data among external validation studies, both retrospective and prospective, was lacking. Only 2 of the 13 retrospective studies we identified reported the race and/or ethnicity of the datasets they used. Among the 37 observational studies and clinical trials, 29 studies reported the age and gender of their study populations, respectively. Only 9 studies reported race and/or ethnicity data. Six of these 9 studies evaluated the EyeArt and IDx-DR models in different settings in the US, Europe, and India.

#### Reference Standards

As with other parameters, clearly defined reference standards is important for the critical assessment of a model’s appropriate use. Almost all observational studies and clinical trials described the reference standards they used to validate their models. The bulk of these studies evaluated models for the diagnosis of DR (28/37), for which clear diagnostic criteria exist.

While we did not compare reference standards between studies with similar intended use, DR studies in particular varied in their use of trained graders, ophthalmologist graders, and reading center graders. Diabetic retinopathy reference standards also varied according to DR grading criteria; studies reported using EURODIAB, international clinical DR, and NDESP among other DR criteria. These differing scales may impact performance metrics and should be considered prior to clinical use. For example, in 1 study comparing the use of a DR classification model against 2 different criteria, DR was diagnosed in 22 patients using EURODIAB versus 73 using international clinical DR classification.^25^

Other ophthalmic conditions, such as glaucoma, are more subjective than DR in terms of diagnosis. In these cases, reference standards may be highly variable and similar models may not be comparable.

#### Performance Metrics

Almost all studies reported the sensitivity and specificity of their models, important for determining clinical utility, and while our intention was not to compare the accuracy of models, we did find a wide range in outcomes for each of the performance metrics we evaluated. Fewer studies explicitly reported PPV and NPV. It is also important to note that while area under the curve is often used as a measure of performance, models evaluated on imbalanced data may appear to perform better than they truly do. In these cases, precision-recall curves, which account for imbalance, may be a better indicator of true model performance.

#### Imageability

Twenty-two of the 37 studies reported the imageability of patients, an important factor for determining the feasibility of integration of models into existing clinical workflows. One study that did not report on imageability continued enrollment until a predetermined number of gradable images was obtained.^23^

#### Scope of Use and Inappropriate Use Cases

All studies reported the intended use of the models they evaluated. However, 15 studies did not state or imply primary users. We judged both of these elements to be important for defining the scope of use. Scope of use was rarely explicitly stated; only 1 study reported the exact scope of use,^14^ while a few others alluded to settings in which the models could be potentially successful. Conversely, most studies did report potentially inappropriate use cases, and cautioned against the use of models without additional evaluation. This information taken together helps define parameters for safe and fair usage.

## Limitations

For all studies included in full-text review, we made every effort to review prior publications regarding models that may detail more information on the training or prior retrospective validation efforts for models. To do so, we relied primarily on citations within included papers. If needed, we also referred to developer websites to elucidate more algorithmic details. Despite our best efforts, however, it is possible that we missed some publications in our search.

Scope of use was a particularly difficult parameter to define and assess. The best example of this parameter was by Abramoff et al in their pivotal trial of IDx-DR. The authors explicitly stated that the model was validated for use in the primary care setting, by health care providers, for the autonomous detection of more than mild DR or DME in adults > 22 years, with diabetes but no prior diagnosis of DR.^14^ Other studies were not as direct in their discussions of scope of use and in those cases, we assessed whether scope of use could be reasonably inferred. For example, in the pivotal trial for EyeArt, the authors report that the system met the predetermined performance end points for detection of referable DR and vision threatening DR, that it could be used autonomously, and that it achieved high rates of imageability with trained operators, in the primary care setting.^16^ Though they did not report specifics about appropriate patients, we were able to infer this based on clearly stated patient inclusion/exclusion criteria and demographic details. Determining scope of use requires an understanding of inappropriate use cases but we chose to evaluate these 2 elements separately. We acknowledge that there is a level of subjectivity in this process.

As the sample size of AI models in ophthalmology that have undergone prospective testing is small, the results of our review may not be generalizable. There may also be bias in the implementation of the selection criteria for studies included and excluded in this review.

Finally, it is important to state that the intent of this review is not to evaluate the quality of the studies or models. For example, we do not comment on whether the reference standards used by the studies are inferior or superior to one another, as this may differ across use cases. We report only whether the authors clearly define what they employ as reference standards. Furthermore, this review is not intended to be exhaustive of all possible measures related to transparency. We evaluate the presence of elements in broad categories and, in many cases, do not further quantify sub-elements. A paper reporting only 1 mechanism for reducing data imbalance (risk-bias mitigating measures) will have fulfilled our evaluation criteria for this parameter in the same way that a study reporting multiple might. This is intentional, as we are not commenting on the effectiveness of such measures, merely whether the algorithm was designed with these concerns in mind and whether the authors report this. We fully acknowledge that authors may have done so without specifically reporting this information in the published articles or supplementary information.

Our aim is to demonstrate the level of transparency in reporting and, though transparency is necessary for producing trustworthy algorithms, it is by no means sufficient. Future systematic reviews and meta-analyses should focus on comparing the performance of models with similar intended uses.

## Conclusions

In this review, we found variable levels of transparent reporting among models that underwent prospective evaluation for ophthalmic conditions. Many models did not report demographic information at the level of model development nor clinical validation. Beyond this, the scope of appropriate use of most models was also ill defined, making many of these models difficult to contextualize. Studies reported between 39% and 87% of the parameters we outlined (Table 3). We do not comment on what constitutes an acceptable percentage, but believe this is an important consideration for regulators to determine.

Only 1 study, Lin 2019, stated use of the CONSORT clinical trial reporting guidelines (published prior to release of CONSORT-AI).^40^ This study reported 23 of 36 of the transparency parameters we outlined.^40^ Two studies used the STARD reporting checklist, and they reported 14 of 36^46^ and 19 of 21^56^ of the transparency parameters we assessed. These relatively low numbers are in part because many of the factors we reviewed were at the level of model development, not only clinical trialing. No studies reported use of the MI-CLAIM checklist, which was published in 2020.^10^ These gaps underscore the need for reporting at the level of model development.

Our search process highlights the need for a central, easily accessible tool for model evaluation and comparison, such as model cards. These cards should be computationally accessible to facilitate digital evaluation. Reviewers, researchers, providers, and patients should be provided with the most up to date and easily accessible information to compare models and decide if they are appropriate for specific populations. Artificial intelligence is already transforming the nature of clinical care and has the potential to improve health outcomes at scale. Reporting standards, both at the level of model development and clinical trials, are essential in ensuring this happens in a fair and equitable way.
