# Supplementary material for: Transparency in Artificial Intelligence Reporting in Ophthalmology-A Scoping Review
Source: Ophthalmol Sci. 2024 Jan 18;4(4):100471. doi: 10.1016/j.xops.2024.100471 (PMC11000111; doi:10.1016/j.xops.2024.100471)
Supplement: Table S1 [file mmc1.pdf]

Supplement Table 1. Search Strategy

Database	Date searched	[complete search strategy]
PubMed	1/13/2022	(("artificial intelligence"[MeSH Terms] OR "algorithms"[MeSH Terms] OR "deep learning"[MeSH Terms] OR "image interpretation, computer assisted"[MeSH Terms] OR "artificial intelligence"[Title/Abstract] OR "machine learning"[Title/Abstract] OR "algorithm"[Title/Abstract] OR "algorithms"[Title/Abstract] OR "deep learning"[Title/Abstract]) AND ("glaucoma"[MeSH Terms] OR "glaucoma"[Title/Abstract] OR "glaucomas"[Title/Abstract] OR ("Macular degeneration"[MeSH Terms] OR "age-related macular degeneration"[Title/Abstract] OR "Macular degeneration"[Title/Abstract] OR "macular degenerations"[Title/Abstract]) OR ("Macular Edema"[MeSH Terms] OR "Macular Edema"[Title/Abstract]) OR ("Keratoconus"[MeSH Terms] OR "Keratoconus"[Title/Abstract]) OR ("corneal dystrophies, hereditary"[MeSH Terms] OR "corneal dystrophy"[Title/Abstract] OR "corneal dystrophies"[Title/Abstract]) OR ("ophthalmology"[MeSH Terms] OR "ophthalmology"[Title/Abstract] OR "ocular surgery"[Title/Abstract]) OR ("Retinal Dystrophies"[MeSH Terms] OR "Retinal Dystrophies"[Title/Abstract] OR "Retinal Dystrophy"[Title/Abstract]) OR ("Myopia"[MeSH Terms] OR "Myopia"[Title/Abstract] OR "nearsightedness"[Title/Abstract]) OR ("anti-vegf"[Title/Abstract] OR "anti-vascular endothelial growth factor"[Title/Abstract]) OR ("Retinal Detachment"[MeSH Terms] OR "Retinal Detachment"[Title/Abstract] OR "Retinal Detachments"[Title/Abstract]) OR ("Retinopathy of Prematurity"[MeSH Terms] OR "Retinopathy of Prematurity"[Title/Abstract] OR "prematurity retinopathy"[Title/Abstract]) OR ("Diabetic Retinopathy"[MeSH Terms] OR "Diabetic Retinopathy"[Title/Abstract] OR "Diabetic Retinopathies"[Title/Abstract])) AND 2011/10/01:2021/10/01[Date - Publication]) NOT "review"[Publication Type]
Medline	1/19/2022	(exp Artificial Intelligence/ or exp Algorithms/ or exp Deep Learning/ or exp Image Interpretation, Computer Assisted/ or "artificial intelligence".tw. or "machine learning".tw. or "algorithm".tw. or "algorithms".tw. or "deep learning".tw.) and (exp Glaucoma/ or "glaucoma".tw. or "glaucomas".tw. or exp Macular Degeneration/ or "age-related macular degeneration".tw. or "macular degeneration".tw. or "macular degenerations".tw. or exp Macular Edema/ or "macular edema".tw. or exp Keratoconus/ or "keratoconus".tw. or exp Corneal Dystrophies, Hereditary/ or "corneal dystrophy".tw. or "corneal dystrophies".tw. or exp Ophthalmology/ or "ophthalmology".tw. or "ocular surgery".tw. or exp Retinal Dystrophies/ or "retinal dystrophies".tw. or "retinal dystrophy".tw. or exp Myopia/ or "myopia".tw. or "nearsightedness".tw. or "anti-vegf".tw. or "anti-vascular endothelial growth factor".tw. or exp Retinal Detachment/ or "retinal detachment".tw. or "retinal detachments".tw. or exp Retinopathy of Prematurity/ or "retinopathy of prematurity".tw. or "prematurity retinopathy".tw. or exp Diabetic Retinopathy/ or "diabetic retinopathy".tw. or "diabetic retinopathy".tw.)<br>Limit to yr "2011 - 2021"<br>Exclude review articles<br>Exclude non-human studies
EMBASE	1/19/2022	(exp Artificial Intelligence/ or exp Algorithms/ or exp Deep Learning/ or exp Image Interpretation, Computer Assisted/ or "artificial intelligence".tw. or "machine learning".tw. or "algorithm".tw. or "algorithms".tw. or "deep learning".tw.) and (exp Glaucoma/ or "glaucoma".tw. or "glaucomas".tw. or exp Macular Degeneration/ or "age-related macular degeneration".tw. or "macular degeneration".tw. or "macular degenerations".tw. or exp Macular Edema/ or "macular edema".tw. or exp Keratoconus/ or "keratoconus".tw. or exp Corneal Dystrophies, Hereditary/ or "corneal dystrophy".tw. or "corneal dystrophies".tw. or exp Ophthalmology/ or "ophthalmology".tw. or "ocular surgery".tw. or exp Retinal Dystrophies/ or "retinal dystrophies".tw. or "retinal dystrophy".tw. or exp Myopia/ or "myopia".tw. or "nearsightedness".tw. or "anti-vegf".tw. or "anti-vascular endothelial growth factor".tw. or exp Retinal Detachment/ or "retinal detachment".tw. or "retinal detachments".tw. or exp Retinopathy of Prematurity/ or "retinopathy of prematurity".tw. or "prematurity retinopathy".tw. or exp Diabetic Retinopathy/ or "diabetic retinopathy".tw. or "diabetic retinopathy".tw.)<br>Exclude non-human<br>Limit to EMBASE<br>Limit to article or article in press<br>Limit to English language
Web of Science	1/20/2022	TS=("artificial intelligence" OR "machine learning" OR "algorithm" OR "algorithms" OR "deep learning") AND TS = ("glaucoma" OR "glaucomas" OR "age-related macular degeneration" OR "macular degeneration" OR "macular degenerations" OR "macular edema" OR "keratoconus" OR "corneal dystrophy" OR "corneal dystrophies" OR "ophthalmology" OR "ocular surgery" OR "retinal dystrophy" OR "retinal dystrophies" OR "myopia" OR "nearsightedness" OR "anti-VEGF" OR "anti-vascular endothelial growth factor" OR "retinal detachment" OR "retinal detachments" OR "retinopathy of prematurity" OR "prematurity retinopathy" OR "diabetic retinopathy" OR "diabetic retinopathies")<br>publication date: 2011-10-01 - 2021-10-01<br>articles
CINAHL	1/20/2022	((MH "Artificial Intelligence") OR (MH "Algorithms") OR (MH "Deep Learning") OR (MH "image interpretation, Computer Assisted") OR TI "artificial intelligence" OR AB "artificial intelligence" OR TI "machine learning" OR AB "machine learning" OR TI "algorithm" OR AB "algorithm" OR TI "algorithms" OR AB "algorithms") AND ((MH "glaucoma") OR TI "glaucoma" OR AB "glaucoma" OR TI "glaucomas" OR AB "glaucomas" OR (MH "Macular Degeneration") OR TI "age-related macular degeneration" OR AB "age-related macular degeneration" OR TI "macular degeneration" OR AB "macular degeneration" OR TI "macular degenerations" OR AB "macular degenerations" OR TI "macular edema" OR AB "macular edema" OR (MH "Keratoconus") OR TI "keratoconus" OR AB "keratoconus" OR (MH "Corneal Dystrophies, Hereditary") OR TI "corneal dystrophy" OR AB "corneal dystrophy" OR TI "corneal dystrophies OR AB "corneal dystrophies" OR (MH "Ophthalmology") OR TI "ophthalmology" OR AB "ophthalmology" OR TI "ocular surgery" OR AB "ocular surgery" OR TI "retinal dystrophies" OR AB "retinal dystrophies" OR TI "retinal dystrophy OR AB "retinal dystrophy" OR (MH "Myopia") OR TI "myopia" OR AB "myopia" OR TI "nearsightedness" OR AB "nearsightedness" OR TI "anti-vegf" OR AB "anti-vegf" OR TI "anti-vascular endothelial growth factor" OR AB "anti-vascular endothelial growth factor" OR (MH "Retinal Detachment") OR TI "retinal detachment" OR AB "retinal detachment" OR TI "retinal detachments" OR AB "retinal detachments" OR (MH "Retinopathy of Prematurity") OR TI "retinopathy of prematurity" OR AB "retinopathy of prematurity" OR TI "prematurity retinopathy" OR AB "prematurity retinopathy" OR (MH "Diabetic Retinopathy") OR TI "diabetic retinopathy" OR AB "diabetic retinopathy" OR TI "diabetic retinopathies" OR AB "diabetic retinopathies")<br>Published Date: 20111001-20211031<br>Research Article
