# Supplementary material for: Transparency in Artificial Intelligence Reporting in Ophthalmology-A Scoping Review
Source: Ophthalmol Sci. 2024 Jan 18;4(4):100471. doi: 10.1016/j.xops.2024.100471 (PMC11000111; doi:10.1016/j.xops.2024.100471)
Supplement: Table S2 [file mmc2.pdf]

Supplement Table 2. Funding Sources

Study ID	Funding
He 2020	This work was supported by Advanced and Appropriate Technology Promotion Project of Shanghai Health Commission(2019SY012), Project of Shanghai Municipal Commission of Health and Family Planning (201740001, 20164Y0180), Science and technology innovation action plan of Shanghai Science and Technology Commission (17411952900), Project of Shanghai Jing'an District Health Research (2016QN06, 2019QN07), Project of Shanghai Jin-g'an District Municipal Commission of Health and Family Planning(2018MS12, 2016084). Project of Shanghai Shibei Hospital of Jing'anDistrict (2018SBMS10)
Bawankar 2017	Funded by Robert Bosch Engineering and Business Solutions Private Limited.Investigators were given grant per completed subject by Think-i,Unit of Tenet Health Edutech Pvt.Ltd.\
Lin 2019	This study was funded by National Key R&D Program of China (2018YFC0116500) and the Key Research Plan for the National Natural Science Foundation of China in Cultivation Project(91846109).
Lin 2021	This study was funded by the National Key R&D Programme of China (2018YFC0116500), the Science and Technology Planning Projects of Guangdong Province (2018B010109008), the National Natural Science Foundation of China (82000946 and 81770967), Natural Science Foundation of Guangdong Province (2021A1515012238), and Fundamental Research Funds for the Central Universities (18ykpy33
Dai 2021	This work was sup-ported by Shanghai Municipal Grants Award (GWIV-3), National Natural Science Foundation of China (NSFC) - National Health and Medical Research Council of Australia (NHMRC) joint research grant (81561128016), Shanghai Belt and Road Joint Laboratory of Intelligent Diagnosis and Treatment of Metabolic Diseases (18410750700),Science and Technology Innovation Action Plan from Shanghai Science and Technology Commission (17411952600) and Shanghai Municipal Key Clinical Specialty to W.J., and General Project from NSFC (61872241) to B.S., and Science and Technology Innovation Action Plan from Shanghai Science and Technology Commission (16DZ0501100) to Q.W
Olvera-Barrios 2021	This work was funded by the North East London Diabetes Eye Screening Programme (RC, LB and JA). This research has received a proportion of its funding (salary support) from the Department of Health's NIHR Biomedical Research Centre for Ophthalmology at Moorfields Eye Hospital and UCL Institute of Ophthalmology (AT and CE), the Lowy Medical Research Institute (TH) and from the Department of Ophthalmology and University Hospital, Universidad Autónoma de Nuevo León (AO-B)
Liu 2020	Supported by the National Eye Institute, National Institutes of Health, Bethesda, Maryland (grant no.: K08EY025269 [R.R.]); and Research to Prevent Blindness, Inc, New York, New York (R.R.). The camera and automated screening software used in the study were provided by Eyenuk Inc.
Ipp 2021	The study was funded in part by grants 9SB1EY027241 and 2R44EY026864 from the National Institutes of Health and in part by Eyenuk Inc. Dr Domalpally was supported in part by an unrestricted grant from Research to Prevent Blindness Inc to the University of Wisconsin Madison Department of Ophthalmology
Rajalakshmi 2018	We acknowledge Dr. Kaushal Solanki, Founder and CEO, EyeNuk Inc.,Los Angeles, California and his team for doing the automated analysis and for providing the technical information about Eye Art software.
Heydon 2020	This research has received a proportion of its funding from the Department of Health's NIHR Biomedical Research Centre for Ophthalmology at Moorfields Eye Hospital and UCL Institute of Ophthalmology. The views expressed in the publication are those of the authors and not necessarily those of the Department of Health. Diabetes prevention research at St George's,University of London, is supported by the National Institute for Health Research(NIHR) Applied Research Collaboration South London (NIHR ARC South London)(grant reference NIHR200152)
Sarao 2020	NR
Van der Heijden 2018	This study was supported by IDx LLC, Iowa City,IA, USA
Wolf 2021	Supported by grants from IDx LLC (Iowa City, IA, USA), Researchto Prevent Blindness (New York, NY, USA). MDA is recipient of theRobert C. Watzke Endowment. This material is the result of worksupported with resources and the use of facilities at the Iowa CityVeterans Affairs Medical Center.
Abramoff 2018	Supported by grants from IDx LLC (Iowa City, IA, USA), Researchto Prevent Blindness (New York, NY, USA). MDA is recipient of theRobert C. Watzke Endowment. This material is the result of worksupported with resources and the use of facilities at the Iowa CityVeterans Affairs Medical Center.
Keel 2018	This work was supported by funding from the Bupa Health Foundation. The authors wish to thank the staff from Barwon Health, Geelong and St Vincent's Hospital, Melbourne endocrinology outpatient clinics for their assistance during this project.
Ming 2021	This work was supported by Henan Key Laboratoryof Ophthalmology and Visual Science and National NaturalScience Foundation of China Grants (No. 82071008,81770949)
Soto-Pedre 2015	This study was supported in part by the DiabetesPlan 2006-10 of Valencia Government Departmentof Health (Valencia, Spain)
Bhuiyan 2021	This project was supported by NIH SBIR Grant: 1R44EY031202
Sosale 2020	Nil.
Sosale 2020	The study was funded by Diacon Hospital and did not receive any external funding
Jain 2020	Nil.
Natarajan 2019	We acknowledge Medios Technologies, Singapore, for providing the AI software for conducting the study.
Rogers 2020	Funding was provided by Visulytix Ltd.* in the provision ofthe Pegasus deep learning software and statistical analysis of results.*Visulytix Ltd. is currently in liquidation
Li 2021	This work was supported by Taichung Veterans General Hospital, Taichung, Taiwan (grantnumber TCVGH-1093502B and TCVGH-1103501D), and National Health Research Institute (grantnumber NHRI-EX109-10927HT)
Zhang 2020	This research was supported by grants from National Key R&D Program of China (2016YFC0901200, 2018YFC1314800); Chinese Academy of Engineering (2019-XZ-42); the National Natural Science Foundation of China (81670797); the Program for Shanghai Outstanding Medical Academic Leader (2019LJ07); the Youth Program of Shanghai Municipal Health and Family Planning Commission (20174Y0081) and the Yang Fan Project of Shanghai Science and Technology Committee (19YF1442700).
Wu 2019	This study was supported by the National Key Research and Development Programme (2018YFC0116500), the Key Research Plan for the National Natural Science Foundation of China in Cultivation Project (91846109), the Science Foundation of China for Excellent Young Scientists (81822010), the National Natural Science Foundation of China (81770967, 81873675, 81800810), the Science and Technology Planning Projects of Guangdong Province (2019B030316012, 2018B010109008, 2017B030314025), Guangdong Science and Technology Innovation Leading Talents (2017TX04R031) and the Natural Science Foundation of Guangdong Province (2018A030310104)
Scheetz 2021	This study was supported by a MRFF RART grant (TA103557) and a JDRF Pilot Initiative Grant (2-SRA-2019–875-M-B). Dr Jane Scheetz is the recipient of a Melbourne Academic Centre for Health Translational Research Fellowship. The Centre for Eye Research Australia receives Operational Infrastructure Support from the Victorian Governmen
Bellemo 2019	National Medical Research Council Health Service Research Grant, Large Collaborative Grant, Ministry of Health, Singapore; the SingHealth Foundation; and the Tanoto Foundation
Porporato 2021	This work was supported by grants from National Medical Research Council and Biomedical Research Council, Singapore (Grant No. 10/1/35/19/674)
Shigueoka 2018	This work was supported by SãoPauloResearchFoundation,FAPESP(07/51281-9).Additional support provided by Novartis,Alcon
Yang 2020	The principal investigator of this study (H Lin) is currently supported by the National Key R & D Project (2018YFC0116500), Fundamental Research Funds of Innovation and Development Project for Outstanding Graduate Students in Sun Yat-sen University (19ykyjs37), the Key Research Plan for the National Natural Science Foundation of China Cultivation Project (91846109), the National Natural Science Foundation of China (81770967), the Fundamental Research Funds for the Central Universities (2018B010109008), and the National Natural Science Fund for Distinguished Young Scholars (81822010)
Kanagasingam 2018	The National Health and Medical Research Council of Australia provided funding for theresearch and development of the machine learning system for diabetic retinopathy.
John 2019	NR
Gulshan 2019	Google Inc sponsored the study. Aravind, Sankara, and EyePACS received fundingfrom Google to support extraction, deidentification,and transfer of images for the study (Dr Kim isaffiliated with Aravind, Dr Raman is affiliated withSankara, and Dr Cuadros is affiliated with EyePACS)
Hong 2021	This study was supported by the National Natural ScienceFoundation of China (81970766 and 8217040684), the Programfor Professor of Special Appointment (Eastern Scholar) atShanghai Institutions of Higher Learning, the ShanghaiInnovation Development Program (2020-RGZN-02033), theShanghai Key Clinical Research Program (SHDC2020CR3052B);LG was supported by the Deutsche Forschungsgemeinschaft(DFG, German Research Foundation), EXC 2026, Cardio-Pulmonary Institute (CPI), Project ID 390649896; and GuizhouScience and Technology Program (GZWJKJ2018-1-003)
Nakahara 2021	This study was supported in part by grants (nos. 19H01114, 18KK0253 and 20K09784 (RA)) from the Ministry of Education, Culture, Sports, Science and Technology of Japan and The Translational Research program; Strategic Promotion for practical application of Innovative medical Technology (TR-SPRINT) from the Japan Agency for Medical Research and Development (AMED) (no grant number), grant AIP acceleration research from the Japan Science and Technology Agency (RA) (no grant number), and grants from the Suzuken Memorial Foundation (no grant number) and the Mitsui Life Social Welfare Foundation (no grant number).
Pawar 2021	Nil.
Long 2017	This study was funded by the 973 Program (2015CB964600), the NSFC (91546101), the Guangdong Provincial Natural Science Foundation for Distinguished Young Scholars of China (2014A030306030), the Youth Pearl River Scholar Funded Scheme (H.L., 2016), and the Special Program for Applied Research on Super Computation of the NSFC-Guangdong Joint Fund (the second phase)
Bhaskaranand 2016	This work was partially funded by NIH grants TR000377 and EB013585
Bhaskaranand 2019	This research has been supported by NationalInstitutes of Health (NIH) grants EB013585 and TR000377.The contents of this publication are solely the responsibilityof the authors and do not necessarily represent the officialviews of the National Eye Institute, the National Institutes ofHealth, or the U.S. government
Niemeijer 2009	This work was supported in part by the National Eye Institute under Grant R01 EY017066,in part by the Dutch Ministry of Economic Affairs under Grant IOP IBVA02016,in part by The Netherlands Organization for Scientific Research (NWO), in partby Research to Prevent Blindness, NY, and in part by the Wellmark Foundation
Abramoff 2016	Supported by grants from IDx LLC (Iowa City, IA, USA), Researchto Prevent Blindness (New York, NY, USA). MDA is recipient of theRobert C. Watzke Endowment. This material is the result of worksupported with resources and the use of facilities at the Iowa CityVeterans Affairs Medical Center.
Hsieh 2021	This work was supported by Taichung Veterans General Hospital, Taichung, Taiwan (grantnumber TCVGH-1093502B and TCVGH-1103501D), and National Health Research Institute (grantnumber NHRI-EX109-10927HT).
Li 2018	This study was supported in part bythe Fundamental Research Funds of the StateKey Laboratory of Ophthalmology, NationalNatural Science Foundation of China (grantno. 81420108008); the Science and TechnologyPlanning Project of Guangdong Province (grantno. 2013B20400003); and the Bupa Health Foun-dation (Australia grant). J.Sh. is supported bya research fellowship from the National Health andMedical Research Council. M.H. receives supportfrom the Research Accelerator Program at theUniversity of Melbourne and from the CERAFoundation. The Centre for Eye Research Australiareceives operational infrastructure support fromthe Victorian State Government, and Research toPrevent Blindness, Inc., provides support to the Stanford University Department of Ophthalmology.
Fu 2019	This work was supportedby ASTAR Biomedical Engineering Programme grant 1521480034 under the Biomedical Research Council and the Singapore Translational ResearchInvestigator Award (NMRC/STAR/0023/2014) from the Singapore Ministry of Health's National Medical Research Council
John 2016	NR
Gulshan 2016	Google Inc was involved in the design and conduct of the study; collection, management, analysis, andinterpretation of the data; and preparation, review,or approval of the manuscript; and decision tosubmit the manuscript for publication.
Shibata 2018	Supported by Grant 17K11418 from the Ministry of Education, Culture, Sports, Science and Technology of Japan and the Translational Research program; Strategic PRomotion for practical application of INnovative medical Technology, TR-SPRINT, from Japan Agency for Medical Research and Development, AMED. This research is (partially) supported by The Translational Research program; Strategic PRomotion for practical application of INnovative medical Technology, TR-SPRINT, from Japan Agency for Medical Research and Development, AMED, Grant 17K11418 from the Ministry of Education, Culture, Sports, Science, and Technology of Japan and Japan Science and Technology Agency (JST) CREST JPMJCR1304
