# Supplementary material for: Transparency in Artificial Intelligence Reporting in Ophthalmology-A Scoping Review
Source: Ophthalmol Sci. 2024 Jan 18;4(4):100471. doi: 10.1016/j.xops.2024.100471 (PMC11000111; doi:10.1016/j.xops.2024.100471)
Supplement: Table S5 [file mmc3.pdf]

Table S5. Imaging Device Manufacturers

| List of Named Models | Study ID | Input type | Camera/Imaging Device |
|---|---|---|---|
| **Airdoc** | He 2020 | Standard Fundus Photo | Topcon TRC-NW400camera |
| **Bosch DR** | Bawankar 2017 | Handheld Fundus Camera Photo | Bosch nonmydriatic fundus camera |
| **CC-Cruiser** | Lin 2019 | Slit Lamp Photo | Not Reported |
| **CARE** | Lin 2021 | Standard Fundus Photo | Multiple<br>Canon (CR2; CR2-AF; CR2-AF plus;CR6-45NM)<br>Topcon (NW 400; NW 300; NW 8; NW 50)<br>Syseye (RetiCam 3100; RetiCam 3000)<br>Crystalvue (FundusVue; TonoVue)<br>Zeiss (VISUCAM 224)<br>Nidek (AFC330)<br>Kowa (VX-20) |
| **DeepDR** | Dai 2021 | Standard Fundus Photo | Canon<br>Topcon<br>Zeiss |
| **EyeArt** | Olvera-Barrios 2021 | Standard Fundus Photo | EIDON Confocal<br>NDESP-approved fundus cameras:<br>AFC-330 Fundus Camera<br>Nidek RS-330<br>Canon CR-2<br>Canon CR-2AF<br>CR-2 Plus<br>CR-2 Plus AF<br>Cirrus 600<br>Cirrus 800<br>iCam<br>Kowa VX-20<br>nonmyd 8 and 8s<br>nonmyd WX-3D<br>NW400<br>TRC NW8 (and F, AF+)<br>DRI OCT Triton (and Plus) |
| | Liu 2020 | Standard Fundus Photo | Canon-CR2 |
| | Ipp 2021 | Standard Fundus Photo | Canon CR-2AF or CR-2 Plus AF |
| | Rajalakshmi 2018 | Smartphone Fundus Photo | Remidio |
| | Heydon 2020 | Standard Fundus Photo | Multiple Cameras |
| | Sarao 2020 | Standard Fundus Photo | TRC- NW8<br>Eidon, Centervue |
| **IDx-DR** | vanderHeijden 2018 | Standard Fundus Photo | TRC-NW100 camera |
| | Wolf 2021 | Standard Fundus Photo | TRC-NW400 camera |
| | Abramoff 2018 | Standard Fundus Photo | TRC-NW400 camera |
| **EyeGrader** | Keel 2018 | Standard Fundus Photo | DRS Centervue |
| **EyeWisdom** | Ming 2021 | Standard Fundus Photo | Canon CR-2 |
| **iGrading** | Soto-Pedre 2015 | Standard Fundus Photo | TRC- NW200 |
| **iHealthScreen** | Bhuiyan 2021 | Standard Fundus Photo | TRC- NW6<br>DRS- Centervue |
| **Medios AI** | Sosale 2020 | Smartphone Fundus Photo | Remedio |
| | Sosale 2020 | Smartphone Fundus Photo | Remedio |
| | Jain 2020 | Smartphone Fundus Photo | Remedio |
| | Natarajan 2019 | Smartphone Fundus Photo | Remedio |
| **Pegasus** | Rogers 2020 | Smartphone Fundus Photo | Pictor Plus Volk Optical |
| **Verisee** | Li 2021 | Standard Fundus Photo | Canon CR-2 |
| **VoxelCloud Retina** | Zhang 2020 | Standard Fundus Photo | TRC-NW400<br>MiiS DSC-200<br>Canon CR-2 PLUS AF<br>Canon CR-2 AF<br>Zeiss VISUCAM20 |
| **List of Unnamed Models** | Study ID | Input type | Camera/Imaging Device |
| **Wu 2019** | Wu 2019 | Slit Lamp Photo | Not reported |
| **Scheetz 2021** | Scheetz 2021 | Standard Fundus Photo | DRS Centervue<br>Canon CR-2 AF<br>Topcon 3D OCT1 Maestro |
| **Bellemo 2019** | Bellemo 2019 | Standard Fundus Photo | DRS Centervue |
| **Porporato 2021** | Porporato 2021 | Anterior Segment OCT | Visante Zeiss |
| **Shigueoka 2018** | Shigueoka 2018 | Clinical data<br>(OCT, SAP parameters) | HVF: Zeiss<br>FDT: Welch Allyn<br>OCT: Cirrus |
| **Yang 2020 (Myopia AI)** | Yang 2020 | External Photos | EVA-AL00<br>Canon EOS 6D<br>iPhone 6<br>iPhone 7 Plus<br>Le X620 |
| **Kanagasingam 2018** | Kanagasingam 2018 | Standard Fundus Photo | Canon CR-2 AF |
| **John 2019** | John 2019 | Standard Fundus Photo | Forus3nethra Classic Non Mydriatic Fundus Camera |
| **Gulshan 2019** | Gulshan 2019 | Standard Fundus Photo | NM TRC<br>Forus3nethra Classic Non Mydriatic Fundus Camera |
| **Hong 2021** | Hong 2021 | Slit Lamp Photo, Standard Fundus Photo | Not reported |
| **Nakahara 2021** | Nakahara 2021 | Smartphone Fundus Photo | D-Eye Lens, Iphone 8 |
| **Pawar 2021** | Pawar 2021 | Portable Fundus Camera Photo | IntucamPrime |
