# Supplementary material for: Transparency in Artificial Intelligence Reporting in Ophthalmology-A Scoping Review
Source: Ophthalmol Sci. 2024 Jan 18;4(4):100471. doi: 10.1016/j.xops.2024.100471 (PMC11000111; doi:10.1016/j.xops.2024.100471)
Supplement: Figure S2 [file mmc4.pdf]

Supplement Figure 2. Suggested Ophthalmologic Model Card

<table>
<tr><td colspan="2">Suggested Ophthalmologic Model Card

Brief Description: an overview of model (e.g. DL model for glaucomatous optic neuropathy screening)
Basic Model Details
– Organization developing model
– Model date/version
– Associated reference/scientific papers for additional information</td></tr>
<tr><td>Intended use
– Primary intended uses (e.g. disease screening, diagnosis, treatment prediction)
– Primary intended users (e.g. ophthalmologists, primary care physician)
– Scope of use (are there populations for which this model is unsuitable? Imaging parameters?)</td><td>Inputs
– e.g. Fundus photos, OCT images or measurements
– Data type e.g. cross-sectional or longitudinal
Outputs
– e.g. Diagnosis, progression, treatment, etc
Model Architecture
– e.g. ResNet CNN, LSTM RNN</td></tr>
<tr><td>Training Data
In proprietary cases, only basic information may be provided
– Dataset source (e.g. clinical trial, telemedicine screening)
– Demographics (e.g. baseline racial/ethnic composition of training data)
– Distribution over various factors (e.g. severities of glaucoma)
– Annotation (e.g. annotated by glaucoma experts, pre-annotated)</td><td>Evaluation Data
Retrospective Validation Data
– Dataset source (e.g. public database)
– Demographics
– Data preprocessing (cropping, image blur, rotation)
– Reference standard (e.g. fundus photo reading center, clinical evaluation)
Clinical Trial Testing
– Demographics
– Environment
– Reference standard</td></tr>
<tr><td>Performance Measures
– e.g. AUC, Sensitivity, Specificity
– Decision thresholds
– Intersectional testing/results? (e.g. performance measures across multiple factors (age, gender, race/ethnicity))

Limitations
– e.g. Image source and quality standards</td><td>Ethical Considerations
What ethical considerations went into algorithm development?
– Risk/bias mitigating measures (e.g. counterfactual testing, testing in diverse settings)
– Potential risks in inappropriate use cases (scope of use, edge cases)
– Input imaging bias risks (e.g. OCT normative database)</td></tr>
</table>
